# Coastal seawater turbidity and thermal stress control growth of reef-building *Porites* spp. corals in Fiji

**DOI:** 10.1038/s41598-025-02283-6

**Published:** 2025-05-17

**Authors:** Ana Samperiz, Sindia Sosdian, Erica Hendy, Kenneth Johnson, Eleanor H. John, Stacy D. Jupiter, Simon Albert

**Affiliations:** 1 School of Earth and Environmental Sciences, Cardiff University, Cardiff, UK; 2 Department of Earth, Ocean, and Atmospheric Science, Florida State University, Tallahassee, FL USA; 3 School of Earth Sciences, University of Bristol, Bristol, UK; 4 Natural History Museum, London, UK; 5 School of Agriculture, Geography, Environment, Ocean and Natural Sciences, University of South Pacific, Suva, Fiji; 6 Global Marine Program, Wildlife Conservation Society, Bronx, NY, USA; 7 School of Civil Engineering, The University of Queensland, St Lucia, Australia

**Keywords:** Nearshore reef, Coral growth, Seawater turbidity, Thermal stress, Fiji, Environmental impact, Marine biology

## Abstract

Nearshore reefs, at the interface of land-sea interactions, provide essential ecosystem services, but are susceptible to multiple global and local stressors. These stressors can detrimentally impact coral growth and the continuity of the reef framework. Here, we analyse coral growth records (1998 – 2016) of massive *Porites* spp. colonies from nearshore reefs in Fiji. Our aim is to assess the role of thermal stress and turbidity on coral growth across a range of environments. Our findings reveal a negative linear relationship between linear extension and seawater turbidity across locations (GLM, R^2^ = 0.42, *p* < 0.001), indicating that average coral growth is significantly influenced by local environmental conditions. On interannual timescales, all locations experienced a 14% to 30% decrease in linear extension in response to acute thermal stress during the 2013 – 2016 period. This finding highlights the existence of compounding effects between water quality and thermal stress. We suggest that inshore, long-lived massive hard corals in areas of high turbidity are more vulnerable to increasing SSTs due to an already reduced mean growth. Integrated management strategies in these regions that considers managing for multiple, interacting local stressors are warranted to enhance resilience.

## Introduction

Coastal and nearshore reefs are structurally complex ecosystems supporting vast levels of biodiversity^1^. The services they provide (i.e. fisheries and material resources, coastal protection, and cultural value and wellbeing^1^) sustain the livelihood of human communities^2^, especially in Small Island Developing States (SIDS)^3^, where more than 94% of people live within 100 km of a reef^4^.

These ecosystems are at the interface of land-sea interactions and, contrary to offshore reefs which are mainly impacted by global stressors, are affected by a range of interactive global and local impacts^5^. At the global scale, thermal stress is the main cause of declining reef-building coral species^6^. However, marine heatwaves are not the sole stressor acting in coastal reefs. At the local scale, human-induced deterioration in water quality and increased sedimentation rates are altering benthic communities and contributing to habitat loss^7^ which, in turn, exacerbate reef-specific response to global events like marine heatwaves^8^. These global and local impacts act synergistically^9^, and affect reef-building corals via reduced coral growth^10^, mass bleaching and mortality^11^, reduced structural complexity^12^ and, ultimately, phase shifts towards reef states dominated by non-calcifying organisms^13^. It is for this reason that management of local risks and impacts on coral reefs remain a key component in increasing ecosystem resilience in the face of climate change^14^.

The effects of both local and global stressors on coastal reefs are complex and highly localised^15^, requiring context-specific reef management. Resilience-based coastal management considers reefs as a coupled social-ecological system^16^. By including conventional ecological metrics (e.g. coral cover ratios, herbivorous fish biomass) with non-traditional resilience-based ones (e.g. coral recruitment, juvenile coral density, algal turf height), better assessments of reef-specific responses to environmental events^17^ can be made. Coral growth rates are another physiological metric that have been considered important for informing resilience-based management, particularly in respect to the capacity of reef systems to recover from disturbance^18^. Untangling environmental drivers of variability in coral growth rates can thus provide a new perspective on the vulnerability of coastal reefs to better inform management.

Scleractinian reef-building corals are key to maintaining the three-dimensional complexity of reefs. However, elevated turbidity from terrestrial runoff^19^, wind and tidal-driven resuspension^20^, and increased pollutants in waters^21^ can lead to low rates of coral growth and photosynthesis, low larval recruitment, and macroalgal competition^22,23^, diminishing the structural diversity of coastal reefs^24^. Nevertheless, the effects of elevated turbidity on reef-building corals are not always negative. In the last decade, turbid reefs have been increasingly studied due to their corals’ resistance to bleaching and mortality during thermal stress^25^, suggesting that some turbid reefs are acting as climate change refugia^26^. Yet, limitations to the thermal resistance of turbid reefs have already been observed due to the increasing incidence of elevated temperature events that surpass suspected thresholds for coral bleaching at these reefs^27^. Further research is needed to assess nearshore reefs across the turbidity continuum under increasing ocean temperatures and a range of local stressors. Most studies to date explore impacts on coastal reefs through coral cover surveys^8^, or experimental manipulative studies (as reviewed by Tuttle and Donahue^28^). Although these studies provide important information about current reef state, they fail to represent other important ecological indicators and do not provide a long-term perspective.

Massive, reef-building corals from the genus *Porites* are widespread across the Indo-Pacific^29^ and are present across a broad range of temperature^30^, salinity^31^, and turbidity^32^ conditions, allowing the study of environmental variability in nearshore reefs and its impact on this taxon. For example, these records enable exploration of past conditions, resilience and long-term responses to multiple compounding global and local stressors in coastal reefs. Several studies have reported declining trends in calcification rates and/or linear extension of *Porites* spp. in inshore reefs from the 1990 s onwards in the Great Barrier Reef^33,34^, Line Islands (Central Pacific)^35^, South-East Asia^36^ and South China Sea^37^ as a result of increasing sea surface temperature (SST) and thermal stress coupled with human pressure and local/reef-specific stressors, although the latter are rarely comprehensively identified. Furthermore, DeCarlo et al.^38^ reported an overall decline in *Porites* spp. calcification across the Indo-Pacific during the last century driven by marine heatwaves decreasing linear extension (rather than density), although with regional variability across environmental gradients that might be caused by differing human impacts, oceanographic and/or climatic regimes. The effects of both global thermal stress and poor coastal water quality on coral growth are documented in the literature, yet local environmental conditions are seldom characterised at high enough temporal and spatial resolution to properly understand drivers of changes in growth and how that could affect carbonate accretion of reefs. It is anticipated that rising sea levels, changes in rainfall patterns and changing land use practices will affect turbidity levels on future reefs^39^. Further, assessing local scale factors for a more informed resilience-based management is critical for SIDS, as their populations are typically highly dependent on reef resources and services, yet they are particularly vulnerable due to limited climate adaptation capacities^40^. Here, we assess the effects of turbidity, SST and thermal stress on the growth rate of *Porites* spp. This information has the potential to improve future predictions of reef accretion and implement better informed reef management strategies.

Fiji, a Pacific SIDS, encompasses over 10% of the total coral reefs in the South-West Pacific (4 550 km^2^^42^) and includes one of the world’s largest fringing systems (the Coral Coast, occurring alongshore the south of Viti Levu for over 80 km^42^). As 76% of the Fijian population live within 5 km from the coast, with many relying directly on thriving coral reef ecosystems for jobs and provision of protein^43^, assessing the trajectory of these ecosystems is key for preserving the services they provide. Some of the extensive coral reef systems within the Fijian archipelago have been identified as potential climate refuges, where future climate impact is expected to be less severe^44^. Yet portions of these same reefs are experiencing considerable threats and impact from local stressors, in particular sediment and nutrient runoff, which are strongly linked to catchment land-use and wastewater discharge^45–47^. Currently, monitoring of Fijian reefs is largely implemented through various types of ad hoc, in situ ecological surveys. These approaches enable observations of changes in biodiversity and coral cover, but their spatial and temporal coverage are limited^41^. Brown et al.^45,46^ modelled the associations between a number of key species and satellite-derived water quality parameters measured from coastal waters off Vanua Levu that were linked to land use and land cover. They found that greater turbidity was associated with lower hard coral cover and lower abundance of certain fish groups, indicating that land-use and local water quality has an important effect on coastal reefs in Fiji. In this context, historical records linking environmental change and reef-building coral growth response can help portray a more comprehensive picture of the current reef state, past conditions and future responses.

**Fig. 1:**
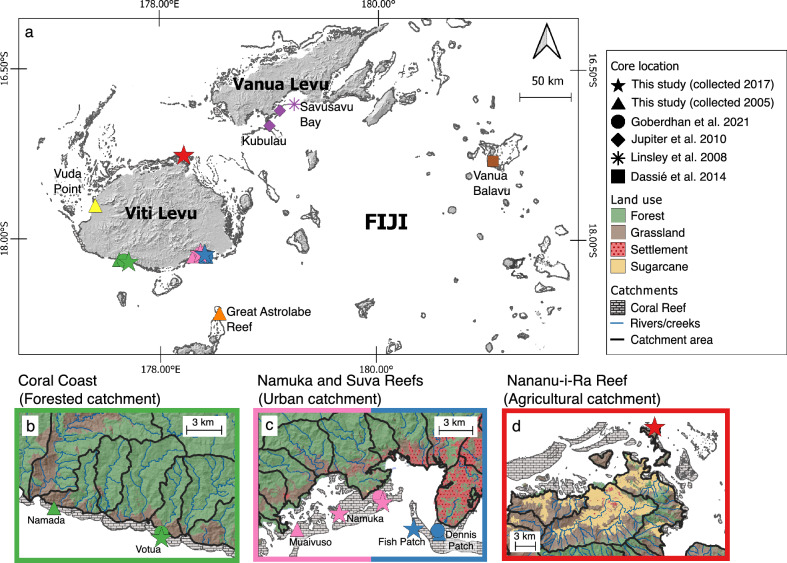
Map of Fiji highlighting the coring locations discussed in this study. Symbols represent sources of sample data (either collected for this study or compiled from the literature). Colours of symbols identify different reefs where samples were collected. Panel **a**) shows part of the Fijian archipelago with all locations of this study. Land is displayed as a digital elevation model (DEM, Source: STRM, 30 m resolution) with a hillshade. Panels **b**), **c**), and **d**) show zoomed-in areas with cores covering the 1998–2016 period. Land is displayed as a DEM overlayed with landcover data, rivers and catchment areas (*Pers. Comm.* Fiji Department of Lands). The maps in this figure were created using QGIS 3.36.3 (https://qgis.org/).

We assess the role of multiple environmental stressors on *Porites* spp. growth in Fijian inshore reefs (Fig. 1) across a turbidity gradient between 1998 and 2016. We present here coral growth histories from 20 *Porites* spp. cores (10 collected in 2005 and 10 collected in 2017) from six locations with different key environmental conditions, namely seawater turbidity, chlorophyll-a concentrations, rainfall, windspeed, SST, thermal stress (measured as degree heat weeks [DHW]) and catchment land cover adjacent to each reef. In addition, we compile existing published records from eight *Porites* spp. covering this same period across six locations in the Fiji Islands^48–51^ to provide the broader context necessary to isolate local controls on growth rates. Below, we use annual data to characterise spatial gradients of coral growth and analyse interannual variability using exclusively core records covering the entirety of the 1998–2016 period (i.e. 11 coral cores). Linear extension was the single metric of coral growth used in this study, as it was available from all published growth records of Fijian *Porites* spp. Although considering exclusively linear extension can limit the observed effects of environmental change on coral growth (e.g. Lough et al.^52^), it is the most consistent metric in our data for between-site comparisons. In addition, calcification in *Porites* spp. is mainly dictated by linear extension^30^, hence supporting its use in this study.

## Results

### Spatial gradients of coral growth and environmental variables

Average linear extension rates for *Porites* spp. corals in each location of this study and their coupled environmental variables are presented in Table 1, Supplementary Table S1 and Supplementary Table S2. Monthly environmental data for the period 1998–2016 are detailed in Supplementary Table S3. In brief, average linear extension rates ranged between 0.78 cm/yr (SD = 0.20) in Nananu-i-Ra (Northern Viti Levu) and 1.44 cm/yr (SD = 0.31) in the Coral Coast (Southern Viti Levu). SST magnitude and seasonality are similar across all locations. Mean annual SST were similar across sites, ranging between 26.81 °C in The Great Astrolabe Reef and 27.82 °C in Vuda Point. (Table 1; Supplementary Table S3; Supplementary Fig. S4). Monthly SSTs warm between September and February (reaching a maximum monthly mean of 28.08 °C in the Coral Coast to 29.33 °C in Vuda Point) and cool between March and August (with a minimum monthly mean of 25.14 °C in Vanua Balavu to 26.17 °C in Vuda Point). Annual K_d_490 (as a measurement of seawater turbidity) and chlorophyll-a concentration recorded at each location presented clear differences in both average values and seasonality profile (Table 1; Supplementary Table 3; Supplementary Fig. S4). Lowest K_d_490 and chlorophyll-a values were found in the Coral Coast (Southern Viti Levu) and Vanua Balavu (offshore island) (0.035 m^−1^ and 0.17 mg/m^3^, and 0.036 m^−1^ and 0.21 mg/m^3^ respectively). The Nananu-i-Ra reef (Northern Viti Levu) registered the highest mean values for K_d_490 and chlorophyll-a (0.070 m^−1^ and 0.51 mg/m^3^). Seasonal profiles of both seawater turbidity and chlorophyll-a in the Coral Coast and Namuka Reef (Southern Viti Levu) record maximum and minimum annual turbidity during December and January and July and August, respectively. Conversely, data from Nananu-i-Ra (and in the Suva Reef to a lesser extent) indicate peak turbidity between December and January, and again between May and June (Supplementary Table S3; Supplementary Fig. S4). Across all sites, K_d_490 and chlorophyll-a data are highly correlated (Supplementary Fig. S6).

**Table 1:** Mean coral growth parameters at each reef and location, with coupled environmental variables for the 1998–2016 period. Coral growth parameters are calculated from annual averages from every core sampled from each location, and include SD. Mean environmental variables are calculated from monthly averages.

Reef	Location	Linear extension		Density		Calcification		Distance to coast	Catch. area	Forest cover	SST	SST summer	K_d_490	Chl-a	Acc. rainfall	Wind speed
		**(cm/yr)**		**(g/cm**^**3**^**)**		**(g/cm**^**2**^** · yr)**		**(km)**	**(km**^**2**^**)**	**(%)**	**(**^**o**^**C)**	**(**^**o**^**C)**	**(m**^**−1**^**)**	**(mg/l)**	**(mm)**	**(m/s)**
Coral Coast	Votua	1.43	(± 0.33)	1.44	(± 0.07)	2.18	(± 0.52)	0.40	10—35	80	27.07	27.87	0.035	0.17	2957.54	2.27
	Namada	1.45	(± 0.57)	1.21	(± 0.12)	1.73	(± 0.52)	0.50	10—35	80	26.91	27.79	0.036	0.18	2957.54	3.23
Namuka	Namuka	1.11	(± 0.22)	1.59	(± 0.05)	1.71	(± 0.22)	1.30	6.5—30	70	26.93	27.86	0.053	0.31	2972.08	3.82
	Muaivuso	1.38	(± 0.31)	1.26	(± 0.05)	1.74	(± 0.26)	1.80	6.5—30	70	26.82	27.81	0.052	0.28	2972.08	3.87
Suva	Fish Patch	0.77	(± 0.11)	1.55	(± 0.05)	1.23	(± 0.12)	2.40	10—20	5	26.93	27.85	0.060	0.41	2936.54	5.35
	Dennis Patch	0.96	(± 0.18)	1.25	(± 0.05)	1.14	(± 0.11)	0.80	10—20	5	26.89	27.81	0.060	0.44	2936.54	5.41
Nananu-i-Ra	Nananu-i-Ra	0.78	(± 0.20)	1.70	(± 0.05)	1.34	(± 0.18)	0.30	20—100	13	27.10	27.95	0.069	0.51	2371.95	6.25
Vuda Point	Vuda Point	1.14	(± 0.31)	1.14	(± 0.06)	1.30	(± 0.20)	2.30	30—200	30	27.82	29.09	0.057	0.36	2939.30	1.75
Kubulau	Kubulau	0.87	(± 0.22)	NA		NA		3.50—10.40	50	70	27.32	28.85	0.062	0.42	1982.91	4.46
Savusavu	Savusavu	1.20	(± 0.12)	1.86	NA	2.23	(± 0.22)	7.30	15—200	10—70	27.36	28.73	0.045	0.21	2363.50	2.87
Vanua Balavu	Vanua Balavu	1.21	(± 0.23)	NA		NA		5.40	50	35	26.85	28.40	0.036	0.21	2257.62	6.41
Great Astrolabe Reef	Great Astrolabe Reef	1.32	(± 0.27)	1.18	(± 0.04)	1.55	(± 0.28)	> 10	0.80	35	26.81	28.35	0.046	0.24	2157.60	6.47

**Fig. 2:**
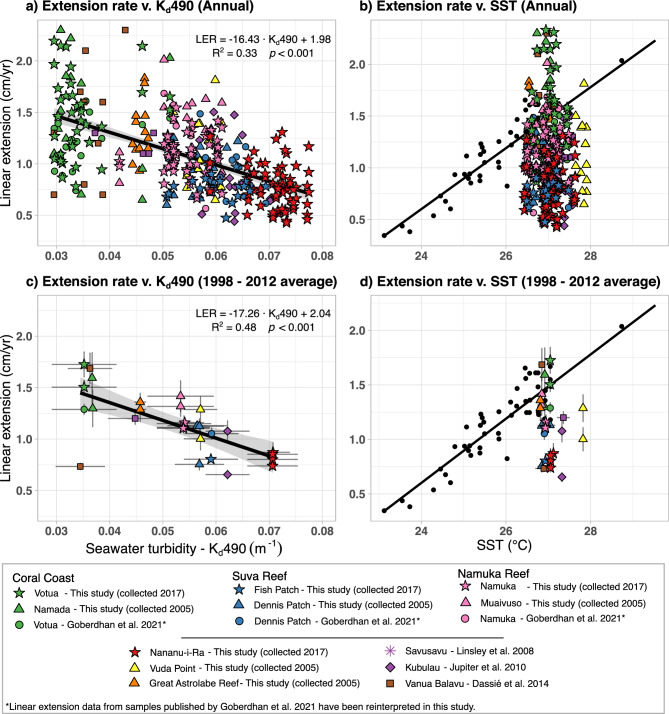
Average linear extension rates of each core. **a**) and **b**): Raw data, core annual linear extension (1998–2016) v. seawater turbidity (measured as K_d_490) and SST, respectively. **c**) and **d**): core average linear extension for the 1998–2012 period (period without thermal stress) v. seawater turbidity and SST. Core samples from the same source and location are represented by the same symbol (e.g. cores VOT17-1 and VOT17-2, both collected from Votua for this study, are represented by a green star). Error bars reflect SE. A significant relationship is only observed between linear extension and seawater turbidity. Black solid lines in **a**) and **c**) represents the linear relationship with 95% confidence interval (grey area). Black datapoints and black line in **b**) and **d**) are extracted from Lough et al. 2014 and represent core average data from *Porites* spp. in the Great Barrier Reef and their relationship with SST.

Linear extension across locations decreased as seawater turbidity increased on both annual timescales and average (calculated excluding the 2013–2016 period due to high thermal stress) (GLM; R^2^ = 0.42,* p* < 0.001; R^2^ = 0.82, *p* < 0.001; Supplementary Fig. S3; Supplementary Table S5). Similar negative linear relationships are observed when averaging by core, instead of by location (GLM; R^2^ = 0.33, *p* < 0.001; R^2^ = 0.48, *p* < 0.001; Fig. 2; Supplementary Table S5). No significant relationship was found between annual average linear extension and SST when averaging by core or by location (GLM, *p* > 0.1; Fig. 2; Supplementary Fig. S3; Supplementary Table S6).

### Interannual variability in coral growth and environmental variables

Interannual variability was assessed only for the 11 coral cores covering the 1998–2016 period, VOT17-1, VOT17-2, CC-LG from the Coral Coast; NAM17-2, NAV17-1, NAV-LG from Namuka Reef; FP17-1, DP-LG from Suva Reef; and NAN17-1, NAN17-3, NAN17-5 from Nananu-i-Ra, to limit potential bias from shorter records. This time period is constrained by the time coverage of seawater turbidity satellite product (SeaWiFS earliest reported data is September 1997 – See Methods).

**Fig. 3:**
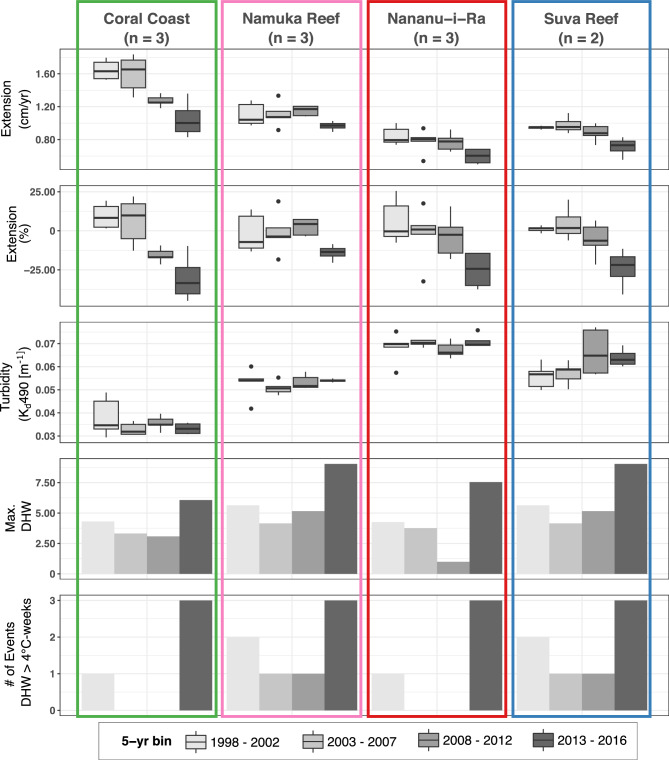
Five-year binned data for absolute (cm/yr) and relative (%) linear extension rates, seawater turbidity, maximum Degree-Heating Weeks (DHW), and number of thermal stress events with DHW superior to 4 °C-weeks recorded at each location. Relative linear extension is calculated as percent change from the average extension at each location during the 1998–2012 period. This analysis only includes records from those cores covering the entire 1998–2016 period (i.e. VOT17-1, VOT17-2 and CC-LG in the Coral Coast; NAM17-2 NAV17-1, NAV-LG in the Namuka Reef; NAN17-1, NAN17-3, NAN17-5 in Nananu-i-Ra Reef; and FP17-1, DP-LG in the Suva Reef).

When data from all locations were assessed in five-year bins, linear extension during the 2013–2016 period was significantly lower than the previous five-year periods present (repeated measurements ANOVA and Tukey post-hoc test, *p* < 0.05) (Fig. 3; Supplementary Tables S7 and S8). Coral linear extension decreased by 31% at the Coral Coast, 25% at Nananu-i-Ra Reef, 24% at Suva Reef and 14% at Namuka Reef, relative to the mean of the 1998–2012 period (years without high thermal stress). When considering data at each location and performing the analysis separately, Namuka Reef showed no significant differences between 2013–2016 and any other period. In Suva Reef, the 2013–2016 period was significantly different to any other time bin. Nananu-i-Ra showed a significant difference exclusively between 1998–2002 and 2013–2016. Additionally, the Coral Coast corals recorded a significant 15% decrease (*p* < 0.05) during 2008–2012 that is not reflected in any other location. In this reef, the 2008–2012 and 2013–2016 are significantly different from the previous periods, but not between themselves.

Using a Generalised Additive Mixed Model (GAMM) (Eq. 1), we demonstrate that observed annual coral growth in Fiji is best explained by including seawater turbidity, thermal stress (as DHW) and SST during the warmest months (Jan, Feb, Mar, Apr) over the studied period (Lowest AIC; R^2^ = 0.57, *p* < 0.05).

1 $$(Linear\ extension = K_{d}490 + SST summer + DHW + s(Core) + s(Core,\ Location))$$

## Discussion

Growth rates in massive *Porites* spp. from nearshore reefs in Fiji are controlled by multiple environmental stressors acting at both the global and local scales. Seawater turbidity plays a significant role in driving average coral growth beyond SST across inshore reefs. However, thermal stress contributes to driving interannual variability with a significant decline in average growth in the last five years. We propose these results highlight the synergistic role of global and local environmental stressors in driving reef growth over space and time.

### Spatial gradients in coral growth are linked to coastal turbidity

The average extension rate of *Porites* spp. in Fiji (i.e. 0.78 cm/yr in Nananu-i-Ra Reef to 1.48 cm/yr in the Coral Coast) is consistent with previous records for this taxa within the Indo-Pacific (~ 0.40 cm/yr measured in the marginal reefs of Hong Kong^53^ to ~ 2.20 cm/yr in the Thai-Malay Peninsula^36^). Environmental conditions such as SST, turbidity and sedimentation rate, light intensity, and nutrient availability, among others have an important influence over annual growth parameters in *Porites* spp. corals^30,32^. As such, the wide range observed in growth records of *Porites* spp. corals (both in Fiji and worldwide) is the reflection of the varying environments where this taxon grows. This is particularly important in inshore reefs, where environmental variability is high and anthropogenic activity causes enhanced exposure to stressors that can hinder coral growth and recovery^54^.

While it has been shown how SST exerts a major control over *Porites* spp. growth^55^, the gradient in the extension rates recorded in Fijian inshore reefs cannot be attributed to SST differences (Fig. 2), as SSTs across all the study locations are similar in terms of absolute values and seasonal profile (Supplementary Fig. S4; Supplementary Table S3). Lough et al.^56^ established an spatial correlation in the GBR with coral growth increasing, on average, 3 mm/yr for each 1 °C increment (R^2^ = 0.84). This relationship has been further exploited to assess whether other environmental parameters beyond SST are driving coral growth in specific locations (e.g. Lough and Cantin^57^, Carilli et al.^35^). In our study, only the cores collected in the Coral Coast (n = 5), Vanua Balavu (n = 2) and the Great Astrolabe Reef (n = 2) fall within the expected growth rates of the above “Coral growth—SST” relationship^56^ based on the local SST, with the average growth from the rest of locations falling below this relationship (Fig. 2). Indeed, the Coral Coast, Vanua Balavu and the Great Astrolabe Reef are all characterised by the lowest seawater turbidity (~ 0.04 m^−1^) and minimal catchment disturbance (> 70% forested). This observation suggests that, in contrast to more impacted sites, local environmental conditions at these reefs are not negatively affecting coral growth. The observed deviation in the relationships between coral growth and local SST in the other locations of this study (i.e. Namuka Reef [n = 6], Suva Reef [n = 4], Nananu-i-Ra [n = 4], Vuda Point [n = 2], Savusavu [n = 1] and Kubulau [n = 2]) is not exclusive to Fiji; a number of studies have reported deviations from the expected relationship in the Indo-Pacific^35,36,58,59^, and suggest that in these locations other environmental parameters (such as turbidity, nutrient concentration or light irradiance) play an important role in coral growth.

It is important to note that Goberdhan et al.^48^ explored growth gradients of *Porites* spp. in inshore reefs of Fiji (four of the locations in this study were also included in their study) and found latitude to be the main driver of coral growth, and not environmental factors. However, their linear extension rates contrast with ours, as they report average growth in the Coral Coast of ~ 0.69 cm/yr (~ 1.44 cm/yr in this study), Namuka Reef ~ 0.85 cm/yr (~ 1.11 cm/yr in this study), and Suva Reef ~ 0.56 cm/yr (~ 0.87 cm/yr in this study). After inspection of Goberdhan et al.^48^ published growth models and core X-rays, we determined that sub-seasonal double banding was counted as single years, substantially underestimating the observed annual growth rates. We reinterpreted data from Goberdhan et al.^48^ (see Methods) to accurate annual extension rates and these disparities across locations were reconciled (Supplementary Table S1).

We report how average coral growth from inshore reefs in Fiji follows a significant negative linear relationship with average seawater turbidity (GLM R^2^ = 0.48, *p* < 0.0001) (Fig. 2). Previous studies have shown that light availability (which is intrinsically linked to seawater turbidity^60^) can play a significant role in controlling average coral growth. However, most of these studies have focused on inshore to offshore gradients as their end members (e.g. D’Olivo et al.^34^), with a lack of detail about local and reef-specific environmental characteristics. Environmental variability in inshore reefs is less studied as isolating individual environmental drivers of coral growth over time is complicated due to the impact of multiple stressors on a range of timescales and their potential synergistic or antagonistic interactions. For example, Lough et al.^52^ analysed growth of *Porites* spp. across 18 locations in NW Australia and found winter SST and photosynthetically active radiation (PAR; also closely linked to seawater turbidity and light availability^61^) to be the primary environmental controls of calcification and linear extension, but a strong inter-correlation between SST and PAR complicated separating their individual contributions to coral growth. The centennial/multi-decadal declines of *Porites* spp. calcification observed by DeCarlo et al.^38^ found regional differences due to varying environmental conditions, with population size and travel distance to reefs used as a proxy measure of local/human pressure from nearby coastal communities. Carilli et al.^35^ looked at growth trends on *Porites* spp. in the Line Islands and found a strong correlation with oceanographic and latitudinal gradients, but also influences from local conditions, although they were not further identified. In the Mesoamerican Barrier Reef System, a steady decline in linear extension of *Siderastrea siderea* since the 1930s has been linked to an increase of river runoff sediment^62^. Similarly, Browne^63^ linked changes in the growth rate of *Turbinaria* spp. in an inshore reef of the Great Barrier Reef to light attenuation. As such, it becomes evident that understanding the interaction between catchment use, local hydrographic factors, turbidity and coral growth underpins management plans at priority reefs.

The mechanisms that can drive seawater turbidity in coastal reefs are diverse and include algal blooms^64^, river runoff^65^, and sediment resuspension and transportation^66^ therefore, reef-specific characterisation is required to understand how local environmental dynamics are impacting coral health. Increased sedimentation in coastal areas has been observed to increase seawater turbidity and limit light availability causing coral smothering and bioerosion^67^, and potentially leading to tissue bleaching and partial mortality^68^. Our results show that sustained seawater turbidity can have other, more subtle, effects over coral physiology, impacting coral growth.

Although sediment and nutrient loads are considered the major pollutants of coastal water in Fiji’s main islands^69^, in situ water quality measurements in coastal reefs are sparse and, in the majority of cases, refer to single measurements^70–72^, complicating the understanding of land-coast interactions on different reefs. Nevertheless, some studies in this same region have linked catchment size and land use^46,73^_,_ as well as human activity^47^ to coastal water quality. This agrees with our observations that reefs near smaller and low-impacted catchments (e.g. Coral Coast, Vanua Balavu and Great Astrolabe Reef) have the lowest turbidity, in contrast to catchments with high anthropogenic activity (e.g. Nananu-i-Ra and Suva Reef). Furthermore, increased turbidity and nutrient loads in Fijian inshore reefs have been identified as the drivers of altered ecological communities^74,75^, reduced coral cover and increased turf and macro-algae^76^. These ecological changes are associated with changes in reef fish communities^45,46^, contributing to long-term ecological shifts and degraded coral reef conditions. Despite the limitations of our study to fully characterise the drivers of coastal turbidity at each of our locations, both remotely sensed water quality and in situ oceanographic data offer insights into how average seawater turbidity at each location is linked to catchment area and land cover and use, and also how seasonal patterns in turbidity differ across locations (Supplementary Fig. S4; Supplementary Table S3). These differing seasonal profiles do not depend on local factors and land use management, but are influenced by climatological components such as rainfall events and changing wind patterns that promote turbulent mixing, and/or oceanic currents and upwelling events, as have been observed in Laucala Bay, near Suva Reef (Southern Viti Levu)^72^ and in Kubulau Reef (Southern Vanua Levu). Consequently, reefs exposed to strong seasonal winds, such as the Nananu-i-Ra and Suva Reefs in this study, are likely to experience enhanced turbidity, potentially explaining why reef-building corals in these regions show lower average growth.

### Thermal stress affects coral growth

**Fig. 4:**
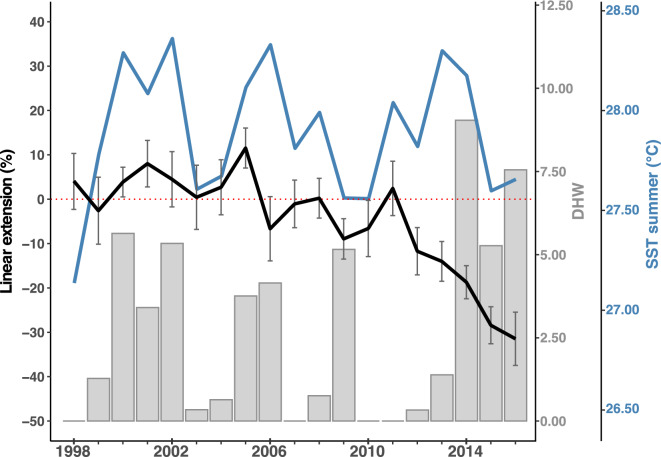
Time-series data of annual coral growth in Fiji for the period 1998–2016. Black line represents normalized average annual change rate in linear extension from all cores collected covering the entire 1998–2016 period (i.e. VOT17-1, VOT17-2, CC-LG, NAM17-2 NAV17-1, NAV-LG, NAN17-1, NAN17-3, NAN17-5, FP17-1, and DP-LG) and it is calculated as percent difference from the 1998–2012 average. Error bars represent SE. Grey columns are maximum observed DHW at all locations. Blue line represents average SST recorded during the climatologically warmest months (Jan, Feb, Mar, Apr).

While on average local seawater turbidity is shown to be a key driver of average coral growth, there is a relationship between region-wide thermal stress and interannual variability of coral growth in Fijian reefs. The five-year bin data analysis of core samples of this study (1998–2016) demonstrates a generalised 14 to 30% decrease in growth across all sites during the 2013–2016 period (Fig. 3). When composing a relative annual growth record of all samples, as shown in Fig. 4, negative growth anomalies are observed in years 2012 and 2013, with a decline of approximately 15%, and a further steep decline between 2014 and 2016, reaching nearly a 30% relative decrease. This decline is partially correlated with exceptionally high thermal-stress levels (DHW > 5 across all sites) persistent during the 2014–2016 El Niño event (Figs. 3 and 4). In addition, no significant changes in seawater turbidity were found in any of the locations over time. The parameters that might affect water quality (i.e. catchment land-use, rainfall events and seasonal winds)^46,73^ have also not experienced significant increases over the studied period^77^_,_ supporting the idea that the observed decrease in growth between 2014 and 2016 was likely caused by thermal stress and not changing local conditions. The 15% decrease observed in 2012 and 2013, however, cannot be attributed to thermal stress, or any other environmental variable included in this study at annual timescales. Further research increasing both the number of samples analysed and the time resolution will aid to better characterise environmental sensitivity of these corals, especially to short-lived sub-seasonal events that might cause a lasting impact on coral growth. As the cores were collected in 2017, following a high thermal stress event in 2016, it is not possible to analyse whether corals across all locations recovered back to pre-stress growth rates, a recovery that has been shown extensively for massive corals in other locations^34,78,79^.

Although coral bleaching is one of the most obvious effects of thermal stress on reef-building corals (e.g. Lough et al.^80^), links between reduced coral growth and diminished reef carbonate accretion in inshore reefs^81^ have also been observed, even before any bleaching evidence^82^. Widespread bleaching events were recorded in Fiji during 2000, 2002, and 2005^76^ but not during the increased thermal-stress period between 2014 and 2016^83,84^. Growth data show a clear link between decreased coral growth and accumulated thermal stress during the 1998–2016 period, despite none of the samples from this study showing evidence of high density bands and/or growth hiatuses commonly linked to thermal stress (e.g. Cantin et al.^78^). This is not unusual as *Porites* spp. are more resilient to thermal stress than many other coral taxa^85^. Yet, this observation suggests that reduced growth and calcification are just one of the multiple responses to thermal stress, these not being as easily identifiable as bleaching^86^.

Discerning the impact of thermal stress in contrast to local environmental conditions on coral growth in Fiji is difficult due to the significant decrease in coral growth that was observed across both sites on the extremes of the turbidity range in this study. The Coral Coast (least turbid site) experienced the highest observed decrease in growth (31%) in respect to the 1998–2012 average. This was followed by the Nananu-i-Ra (most turbid site) and Suva Reefs (~ 25%). This is in contrast to abundant evidence that identifies the role that local anthropogenic stressors (overfishing, eutrophication, increased sedimentation) play in diminishing the thermal threshold of corals, potentially leading to bleaching and compromised annual calcification rates^10,35^. Interestingly, other studies have explored the role of turbid reefs as climate refugia, where suspended sediments limit light irradiance, thus alleviating thermal stress, limiting coral bleaching^81,87^ and facilitating coral heterotrophy^58^. These studies found a genus-specific effect, with *Porites* spp. still showing signs of stress despite elevated turbidity^81^. This benefit occurs in coastal reefs in a turbidity ‘goldilocks’ zone with K_d_490 values between 0.08 and 0.13 m^−1^^87^ (our most turbid reef, Nananu-i-Ra, has an annual average value of 0.07 m^−1^), and with turbidity driven by tidal regime^81^, as opposed to reefs with ephemeral turbidity events driven by accumulated rainfall, winds and/or upwelling currents (as in the locations of this study). Overall, our results support the idea that the existence and nature of compounding effects between water quality and thermal stress is complex and highly reef-specific.

While the differences in relative decline of coral growth during thermal stress were partially influenced by water quality, with higher relative decrease in the Coral Coast (least turbid), absolute growth rates were significantly lower in the Nananu-i-Ra Reef (most turbid). We hypothesize that the impacts of increasing SSTs and thermal stress are more pronounced in the more turbid sites due to an already lowered linear extension, but recognise the need to consider density (and calcification) to obtain a full picture of coral growth. Our hypothesis agrees with studies focusing on the interaction of thermal stress and local environmental conditions on calcification rates of individuals over time^34^ and general reef carbonate production^81^. Although the role of thermal stress in reducing coral growth rates is clear, long-term historical records (on the scale of centuries) are required to establish the extent to which coastal water quality may be altering the resilience of Fijian reef-building corals.

Marine thermal stress^86^ is becoming more prevalent and as limiting global warming to 1.5 °C by the end of the century becomes unlikely^88^, pressures on coral reefs are expected to increase. In Fiji, where corals already live within 2 °C of their upper thermal tolerance^89^, SST is predicted to increase between 1.3 and 2.4 °C by the end of the century^77^. Increased long-term acclimatization of coral populations through successive exposure to thermal stress has been observed in some areas of the Indo-Pacific (e.g. Fox et al.^90^ Oliver and Palumbi^91^), and some Fijian reef sites have already been identified as having higher thermal resistance than other regions^83,86^. Yet, local water conditions have been found to modulate the coral holobiont response and adaptation to thermal stress^92^. Thus, the continuity of these reefs will critically depend on our capability to manage local stressors as a means to enhance coral resilience. Similarly, accumulated rainfall during the wet season is also projected to increase over the course of the twenty-first century in the region, and extreme rainfall events and tropical storms are predicted to be more intense and frequent^77^. Consequently, increased sediment input and elevated turbidity may become more common in nearshore reefs, which will further limit coral growth rates. Unfortunately, the potential for local reef management strategies in Fiji is limited and reef-specific because, as we demonstrate in this study, the seawater turbidity in Fijian inshore reefs does not depend exclusively on local factors and land use management, but it is amplified by rainfall events and seasonal wind patterns. As a result, the observed intensification in the Pacific trade winds in the last three decades^93^ could also have important repercussions on reef turbidity in Fiji, and subsequently on coral growth rates.

It is therefore evident that reef-building coral taxa in inshore areas are impacted by both local seawater turbidity and thermal stress. Furthermore, these stressors in Fiji reefs are not only a major driver of coral cover^45^, but also on coral growth. Land use policies aimed at limiting river runoff and sedimentation to sustain coral growth can potentially be a successful management strategy for some of the inshore reefs less exposed to seasonal winds and wind-driven turbidity in the framework of the current ocean warming and increasing thermal stress events, although a location-specific management approach will still be required. Overall, the results demonstrate that reef management cannot occur in isolation of potential disturbance from non-reef-specific factors, like rainfall events and seasonal winds. These findings support efforts^94–96^ implemented by reef managers in Fiji and other SIDS working to establish an integrated management approach towards reducing coastal turbidity and therefore the potential to improve coral growth rates, maintaining coral cover and sustaining a healthy and productive reef. However, expanding knowledge of coastal and land-use processes is required to pinpoint the drivers of increased turbidity in each location, such as sediment load and nutrient concentration, which can lead to algal blooms, sourced by river runoff, driven by wind-mixing, or upwelling.

## Conclusions

Our results show that seawater turbidity impacts *Porites* spp. growth across inshore reefs in Fiji. Simultaneously, on interannual timescales, thermal stress led to a decrease in coral growth despite differences in background environmental conditions. Specifically, a significant decrease in coral growth was observed in Fijian inshore reefs between 2014 and 2016 because of enhanced thermal stress. This suggests that resilience of reef-building *Porites* spp. corals in Fijian inshore reefs to thermal stress is not just linked to local water quality. Both thermal stress and seawater turbidity are contributing to the increased vulnerability of inshore reefs, but the pathways by which they affect coral growth are different. These stressors are enhanced by both local and global scale parameters, and this suggests that local mitigation through integrated reef management might only be useful in those areas that are not predicted for future increases in turbidity via enhanced wind and rainfall. Inshore reefs and their vulnerability to environmental changes is not uniform across spatial scales, and as such, effective management needs to be adapted to each local reef.

## Methods

### Sample locations

The Republic of Fiji, situated between 15° and 22°S latitude and 174°E and 177°W longitude, includes more than 300 islands, 500 cays and 1 000 coral reefs, with a coastline covering approximately 1 130 km (Fig. 1). The two main islands, Viti Levu (10 386 km^2^) and Vanua Levu (5 535 km^2^), represent 87% of the total land area of Fiji. In 2017, the population of Fiji was 884 887, where 81% and 15% inhabited Viti Levu and Vanua Levu, respectively^97^.

The climate of Fiji is characterised by two differentiated seasons, from November to April (wetter/summer) and May to October (drier/winter)^98^. The seasonality is influenced by the South Pacific Convergence Zone (SPCZ), which lies north of Fiji and fluctuates northeast and southwest, being closest to Fiji in the wet season^77,98^. The southeast trade winds are prevalent in the Fijian archipelago through the year and typically become stronger during the drier/winter season^72^. Rainfall in Fiji is highly variable, and it is influenced by island topography and the dominating trade winds^98^, causing the southeast side of the islands to be more humid and to experience higher annual rainfall than the northwest. Interannual climatic variations are mostly influenced by ENSO, with El Niño events being drier and cooler than normal, and the opposite conditions during La Niña^77^.

In total, we analysed coral growth records from 28 *Porites* cores collected in twelve different locations across Fiji (Fig. 1; Table 1; Supplementary Table S1). Ten coral cores were drilled in May 2017 from live *Porites* spp. colonies from four different inshore reefs around the coast of Viti Levu. Two of the cores were collected from Votua Reef, along the Coral Coast (Southern Viti Levu), three cores were collected from Namuka Reef (Southern Viti Levu), one core was collected from Fish Patch at Suva Reef (Southern Viti Levu), and four cores were collected from Nananu-i-Ra Reef (Northern Viti Levu). The top of the colonies sampled was measured at a depth of 2 m below the seawater surface (except the *Porites* spp. colony at Fish Patch in Suva Reef, which was at a depth of 8 m). These colonies were approximately between 1 and 2 m in height. Coral cores were extracted from the top of the colonies using a pneumatic drill fitted with a 6 cm diameter drill barrel. Coral core cavities were filled with resin plugs to minimize impact to coral colonies. All the coral cores were immersed in fresh water for 24 h and consequently air dried for several days. Ten *Porites* spp. cores were collected in November 2005. Of these, eight were from four inshore reefs in Viti Levu (two cores from Vuda Point [Western Viti Levu]; two cores from Namada Reef [Coral Coast, Southern Viti Levu]; two cores from Muaivuiso Reef [Southern Viti Levu]; and two cores from Dennis Patch at Suva Reef, [Southern Viti Levu]). Also, two cores were collected from a fringing reef in Dravuni Island, located along the Great Astrolabe Reef, an extensive offshore reef system in southern Fiji. Colonies were between 1 and 3 m in height and were cored from the top of the colony using a pneumatic drill with a 6 cm diameter drill barrel.

To increase sample size and validate our observed growth rates, we also included data from other published *Porites* spp. core records from Fiji (Supplementary Table S1). These included: i) three core records from four locations in Southern Viti Levu (i.e. Coral Coast, Namuka Reef and Dennis Patch/Suva Reef) collected in 2018^48^ ii) two core records from two reefs in Kubulau District (Southern Vanua Levu) collected in 2006^49^ iii) one core from an inshore reef in Savusavu Bay (Southern Vanua Levu) collected in December 2001^50^ iv) and two cores from Vanua Balavu Island^51^ collected in 2004. We used the published growth rates from all of the above, except for those of Goberdhan et al.^48^ due to disparities in linear extension between cores within the same locations. Goberdhan et al.^48^ incorrectly assigned sub-seasonal double banding as annual extension records, leading to much lower extension rates than those observed here. Upon accessing the X-rays of coral cores in Goberdhan et al.^48^, we obtained new coral age models for their samples and used those in our analyses. For example, average growth for the sample cored in the Coral Coast (CC-LG) was 0.69 cm/yr, and after reinterpreting X-rays of this same sample, we found it to have an average growth of 1.38 cm/yr. The newly obtained coral growth rates had a good agreement with other samples from the same locations (Supplementary Figure S8 and Supplementary Table S1).

### Environmental data

In this study we use available instrumental environmental data (i.e. SST, rainfall, K_d_490, chlorophyll-a, rainfall and wind speed) for the period between 1998 and 2016. Mean environmental values for each location are described in Table 1 and monthly data from 1998 until 2017 are presented in Supplementary Figure S4 and Table S3. A full description of the environmental data can be found in Supplementary Text S1.

In brief, daily SST data were derived from OISST (v. 2.1)^99^ satellite product by selecting the cell (0.25° × 0.25°) containing each core location. Satellite-derived SSTs were validated using in situ seawater temperature data obtained from the Reeftemps network dataset^100^ (Supplementary Text S1 and Supplementary Fig. S5). Annual average SSTs were calculated as the arithmetic mean of daily data. Summer average SSTs were calculated as the arithmetic mean of daily SSTs during the climatologically warmest months of the year (i.e. January, February, March, April). SST anomalies were obtained by calculating the difference between composite SSTs (annual or summer averages) and SST climatology for the studied period (1998–2016) for either annual or summer-month intervals. Five-year binned data were calculated as the arithmetic mean of annual SSTs in intervals of five years.

Thermal stress was also evaluated as Degree Heating Weeks (DHW). DHW were obtained from the CRW *CoralTemp*’s daily global 5 km satellite coral bleaching DHW^101^. Daily DHW data was obtained from the pixel containing each reef location (25 km^2^). For analysis of data in yearly intervals (annual data), we used the maximum DHW recorded for each year. Five-year binned data were calculated as the maximum DHW recorded for each five-year interval. In order to also consider potential cumulative stress through several years within each bin, we also calculated the number of thermal stress events with DHW equal or higher to > 4.0 °C-weeks. This threshold is sourced from NOAA CRW methodology as the 1^st^ level or bleaching warning, although recent studies call for the need of region-specific thresholds that are iteratively refined^102^.

To characterise water quality at each location, diffuse attenuation coefficient at the wavelength of 490 nm (K_d_490 [m^−1^]) and chlorophyll-a concentration (mg/m^3^) were derived from satellite ocean colour (OC) data. Average daily level 3 data were acquired from SeaWiFS^103^ (9 × 9 km resolution) for the period 04/September/1997 to 03/July/2002, and from MODIS-Aqua^104^ (4 × 4 km resolution) for the period 04/July/2002 to the present. An area of 1 × 1 pixels for SeaWiFS and 2 × 2 pixels for MODIS-Aqua (81 km^2^ and 64 km^2^ respectively) were selected and averaged around each reef location. Annual averages of both parameters were calculated as the arithmetic mean of the daily data from each year. Five-year binned data were calculated by averaging annual means in five-year intervals. Although in the results we report average values of both K_d_490 and chlorophyll-a concentration, we use only K_d_490 for building statistical models due to the close correlation between these two parameters (R^2^ = 0.93) across all locations of this study (Supplementary Fig. S6).

Daily rainfall and wind direction/speed data at the closest available weather stations to each study site were provided by the Fiji Meteorological Service. Rainfall data were available from 1989 until present only for the Navua, Laucala Bay and Rakiraki weather stations. The availability of wind data differed across weather stations, with the longest database available from Nadi airport (from September 2004 to the present), while the rest of the records ranged between 2011–2015 to the present. Land use data from each island were provided by the Fiji Department of Lands.

### Quantification of coral growth parameters

Growth parameters from coral cores collected in May 2017 were extracted from X-ray computed tomography (CT) scans. To derive absolute density values, a hydroxyapatite density standard was scanned together with each core. This standard has five inserts with differing density (i.e. 1.26 g/cm^3^, 1.44 g/cm^3^, 1.65 g/cm^3^, 1.77 g/cm^3^, and 1.92 g/cm^3^) that allowed to build a “grey value – density” calibration curve. Analysis were carried out at the Natural History Museum of London imaging facilities on a Nikon Metrology HMX ST 225 with a resolution of 50 μm. The obtained CT scans were examined to identify the orientation of the major growth axis, which guided optimum tracks to measure coral growth parameters. Linear extension rates were extracted from density profiles of each coral core, measuring distance between density maxima. Skeletal density was measured continuously along the core length from the CT imaging. A virtual segment was established following the major growth axis along the length of the core, avoiding areas of distorted growth (growth scars, bioerosion, growth valleys). A virtual square (5 × 5 mm) was drawn perpendicular to the segment (and therefore to the major growth axis) along all the slices of the coral core. The area of this square was chosen to be able to obtain a density value averaged across at least 20 corallites, but without extending towards a skeletal area secreted at a different period due to bumpy growth of most cores. Each voxel’s grey value enclosed within this area was measured and averaged. Voxel refers to a three-dimensional “pixel” in CT scans. Following the CT image resolution, a single grey value was obtained every 50 μm along the core length, and then converted into density data (mg/cm^3^). The calcification rate (g CaCO_3_/cm^2^ yr) was also obtained as a product of linear extension and skeletal density.

Coral cores collected in November 2005 were sliced lengthwise using a diamond-tipped circular saw into 7 mm thick slabs at the Australian Institute of Marine Science (AIMS) and X-rayed at the Wesley Hospital (Townsville, Australia). Density measurements along the major growth axis were obtained from a combined X-ray densitometer/luminometer. Annual linear extension was obtained measuring distance density maxima by using Coral XDS software^105^.

Only linear extension data was available from the cores collected in Kubulau (Vanua Levu)^49^. These cores were cut into ~ 7 mm thick slabs and X-rayed at Australian National University (ANU) and the core chronologies were established by measuring distance between high-density bands.

Linear extension data from *Porites* spp. samples in Goberdhan et al.^48^ were obtained by first slicing the coral cores in ~ 7 mm slabs and then taking X-rays of the slabs at the College of Medicine, Nursing and Health Sciences Department of Medical Imaging Science in Suva, Fiji. Annual extension was measured as the distance between consecutive high-density bands. Refer to Supplementary Fig. S8 for more information on the reinterpretation of X-rays and growth records from these coral cores.

For the *Porites* spp. sample from Savusavu Bay (Vanua Levu)^50^, annual linear extension rates were obtained from δ^18^O and Sr/Ca data, by measuring the distance between signal minima and maxima and tying points with SST data^50^. Density measurements were carried out by CT scanning the coral slabs at the Albany Advanced Imaging Center, New York. Average density was measured at five-year intervals over an area approximately covering 2 years of growth^106^.

Finally, only linear extension rate data are available from the coral cores from Vanua Balavu. These were obtained from δ^18^O measurements along the major growth axis, by linking the lightest δ^18^O in each seasonal cycle to the warmest month of the year and measuring the distance between these points^51^.

For our compilation, to maximize sample size, we use annual linear extension rates as a measurement of coral growth rather than coral calcification. While coral calcification can be considered a more comprehensive measurement of coral growth (as it is calculated from both linear extension and density), *Porites* spp. data shows a strong correlation between calcification and linear extension (R^2^ > 0.90; Supplementary Fig. S2), indicating that linear extension can represent growth in this genus^30^. In addition, not all available *Porites* spp. cores have density measurements, but for all of them it was possible to obtain annual extension data. Relative linear extension was calculated only for those cores covering the entire 1998 to 2016 period by normalising single annual extension to the mean over the 1998–2012 period (reported as % change). This 1998–2012 period was selected to avoid growth anomalies as a result of thermal stress. Relative growth changes per location were calculated by obtaining the arithmetic mean of relative growth from all cores from a given location. Composite relative growth for Fiji was obtained by applying the arithmetic mean to the annual relative growth of all the cores. Five-year binned data were calculated as the arithmetic mean of annual relative growth in intervals of five years.

### Statistical modelling

Generalised Linear Models (GLM) were used to examine the relationship of SST, thermal stress (both summer SST and DHW) and seawater turbidity with annual growth data. Five-year binned data were calculated to allow for high interannual variability in growth rates. A repeated measures ANOVA test was used to identify reef-specific significant changes in growth during each time period (Supplementary Tables S7 and S8). We used Generalised Additive Mixed Models (GAMM) to examine the relationship between each coral growth and the local environmental variables during the period 1998–2016, following methods in Cooper et al.^107^. Different models were tested (Supplementary Table S9) where Linear Extension was the dependent component, and local environmental variables (i.e. Seawater turbidity, DHW, SST, SST summer, wind speed, accumulated rainfall) were used as the fixed components of the model. The models tested included random effects [i.e. “s(Core)” and “s(Core, Location)”] to include differences in growth between specimens and the interaction between growth of samples at each location in order to limit auto-correlation and the non-independency of these data variables. Due to the nature of the samples, the model also included a first-order correlation to account for the non-independency of measurements within each core and limit auto-correlation. To find the best fitting GAMM model that could explain coral growth variability we used Akaike Information Criterion (AIC). All statistical analyses were performed in R.

## Supplementary Information

Supplementary Information 1.

Supplementary Information 2.

## Data Availability

All data necessary to reproduce the analysis of this study are available in the Supplementary Information. This same data are also available via Zenodo (10.5281/zenodo.15397405).
