# Supplementary material for: Coastal seawater turbidity and thermal stress control growth of reef-building Porites spp. corals in Fiji
Source: Sci Rep. 2025 May 17;15:17172. doi: 10.1038/s41598-025-02283-6 (PMC12085587; doi:10.1038/s41598-025-02283-6)
Supplement: Supplementary file 1 — Supplementary Information 1. [file 41598_2025_2283_MOESM1_ESM.pdf]

Supplementary information for

Coastal seawater turbidity and thermal stress control growth of reef-building *Porites* spp. corals in Fiji.

Ana Samperiz[1,2,*], Sindia Sosdian[1], Erica Hendy[3], Kenneth Johnson[4], Eleanor H. John[1,5], Stacy D. Jupiter[6], Simon Albert[7]

[1]School of Earth and Environmental Sciences, Cardiff University, Cardiff, UK; [2]Department of Earth, Ocean, and Atmospheric Sciences, Florida State University, Tallahassee, FL, USA; [3]School of Earth Science, University of Bristol, Bristol, UK; [4]Natural History Museum, London, UK; [5]School of Agriculture, Geography, Environment, Ocean and Natural Sciences, University of South Pacific, Suva, Fiji; [6]Melanesia Program, Wildlife Conservation Society, Suva, Fiji; [7]School of Civil Engineering, The University of Queensland: St Lucia, AU

*Corresponding author: asamperizvizcaino2@fsu.edu

Content of this file

Supplementary Figure S1. Annual linear extension data from all the cores used in this study for the period 1998 – 2016.

Supplementary Figure S2. Relationship between average growth parameters for 21 cores with linear extension and density data included in this paper for the period 1998 – 2016.

Supplementary Figure S3: Average linear extension at each location versus seawater turbidity and SST.

Supplementary Figure S4. 1998 – 2016 monthly mean data for all environmental variables at every location.

Supplementary Figure S5. OISST data validation with *in situ* seawater temperature loggers.

Supplementary Figure S6. Linear correlation between daily Chlorophyll-a and K_d490.

Supplementary Figure S7. Scatterplot of wind speed data from available local weather stations (*in situ*) and remotely sensed data from CCMP product.

Supplementary Figure S8. Age model correction from samples in Goberdhan and Kinninmonth (2021).

Supplementary Text 1: Source and methods for obtaining environmental data.

Supplementary Tables S1 to S9 are available on attached Excel file (“Tables.xlxs”).

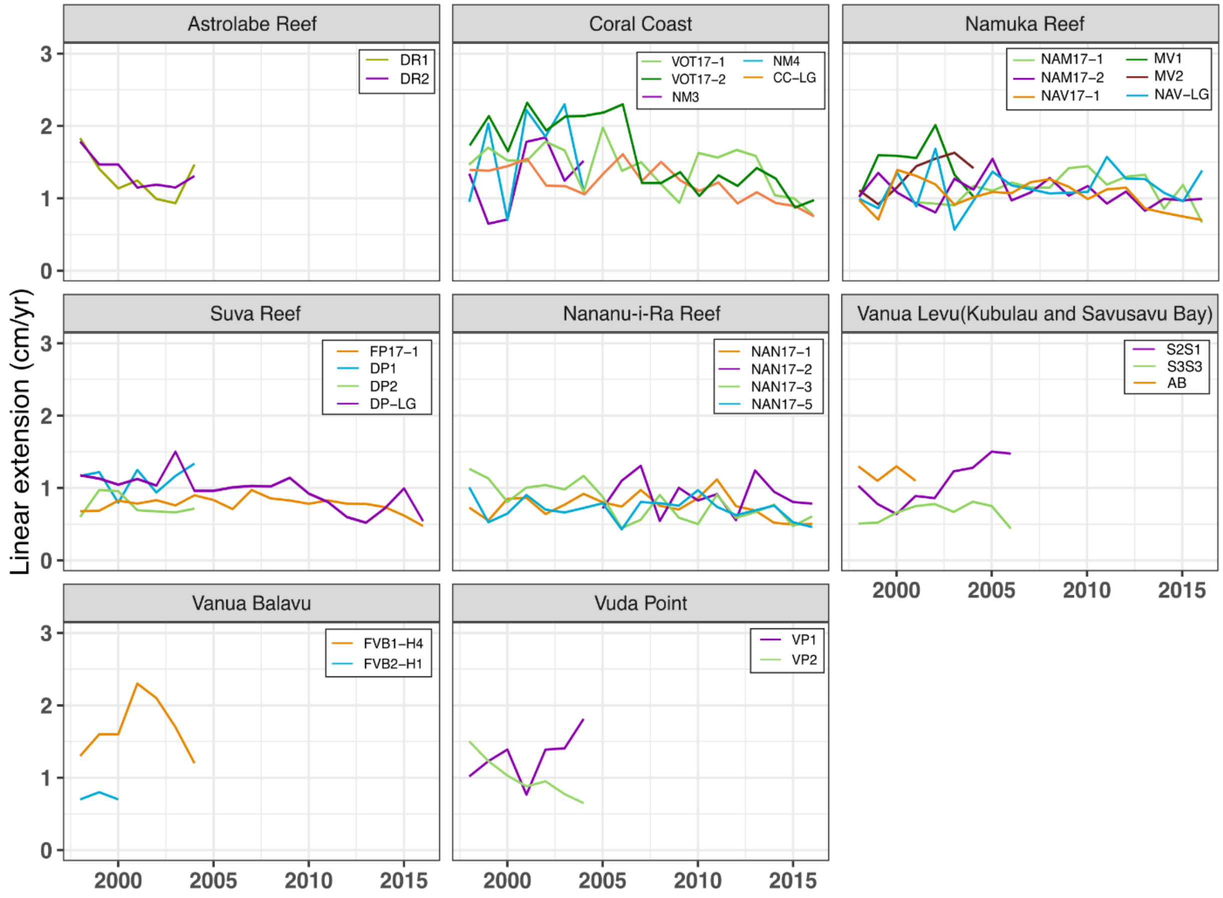

Supplementary Figure S1. Annual linear extension data from all the cores used in this study for the period 1998 – 2016.

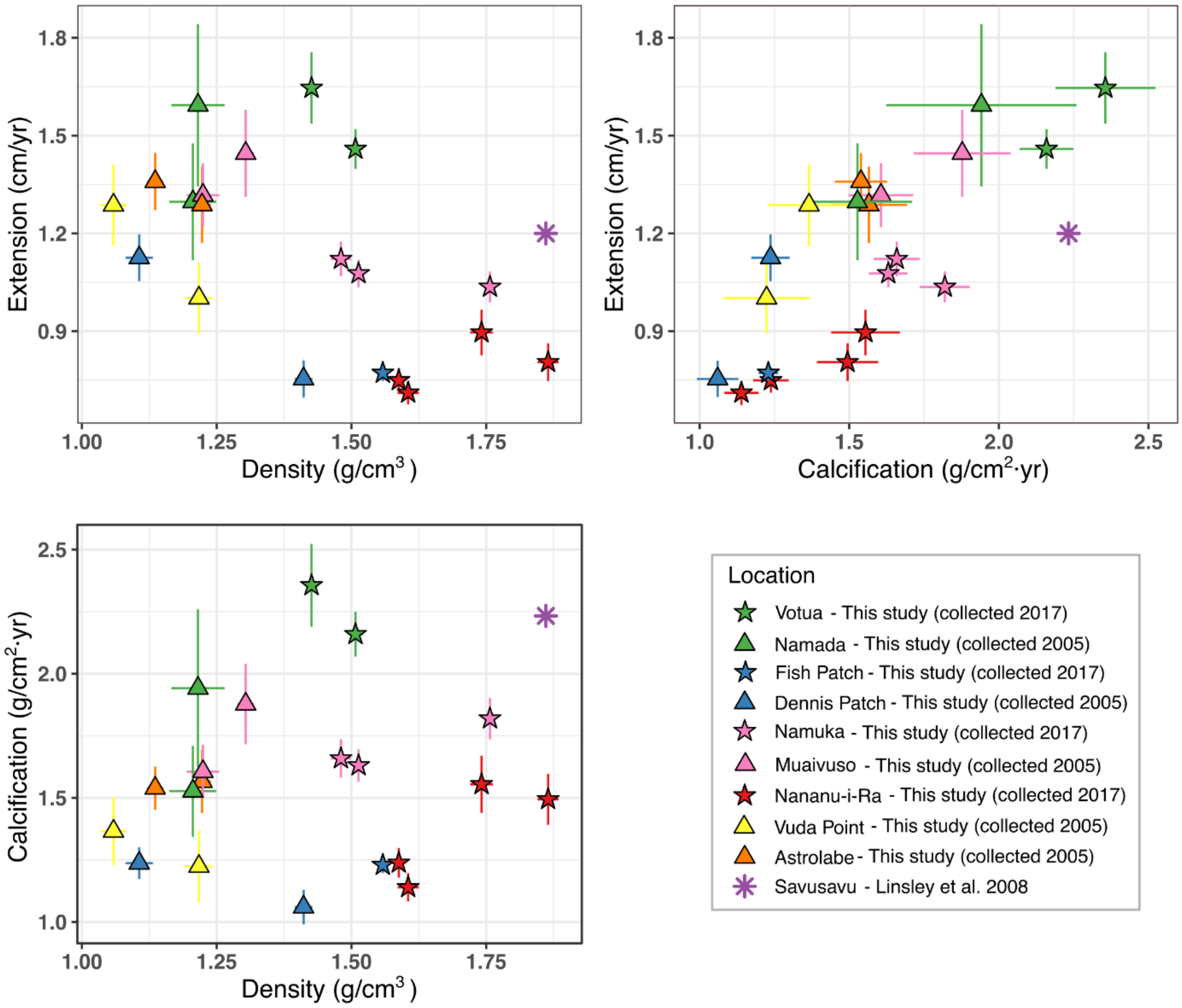

Supplementary Figure S2. Relationship between average growth parameters for 21 cores included in this paper for the period 1998 – 2016. Includes only those sample cores with both linear extension and density data available. Density of samples of this study collected in 2005 (triangle symbols in figure) were obtained via X-ray densitometer, density from samples collected in 2017 (star symbols) and from Linsley et al. 2008 sample (asterisk symbol) were obtained via CT scanning.

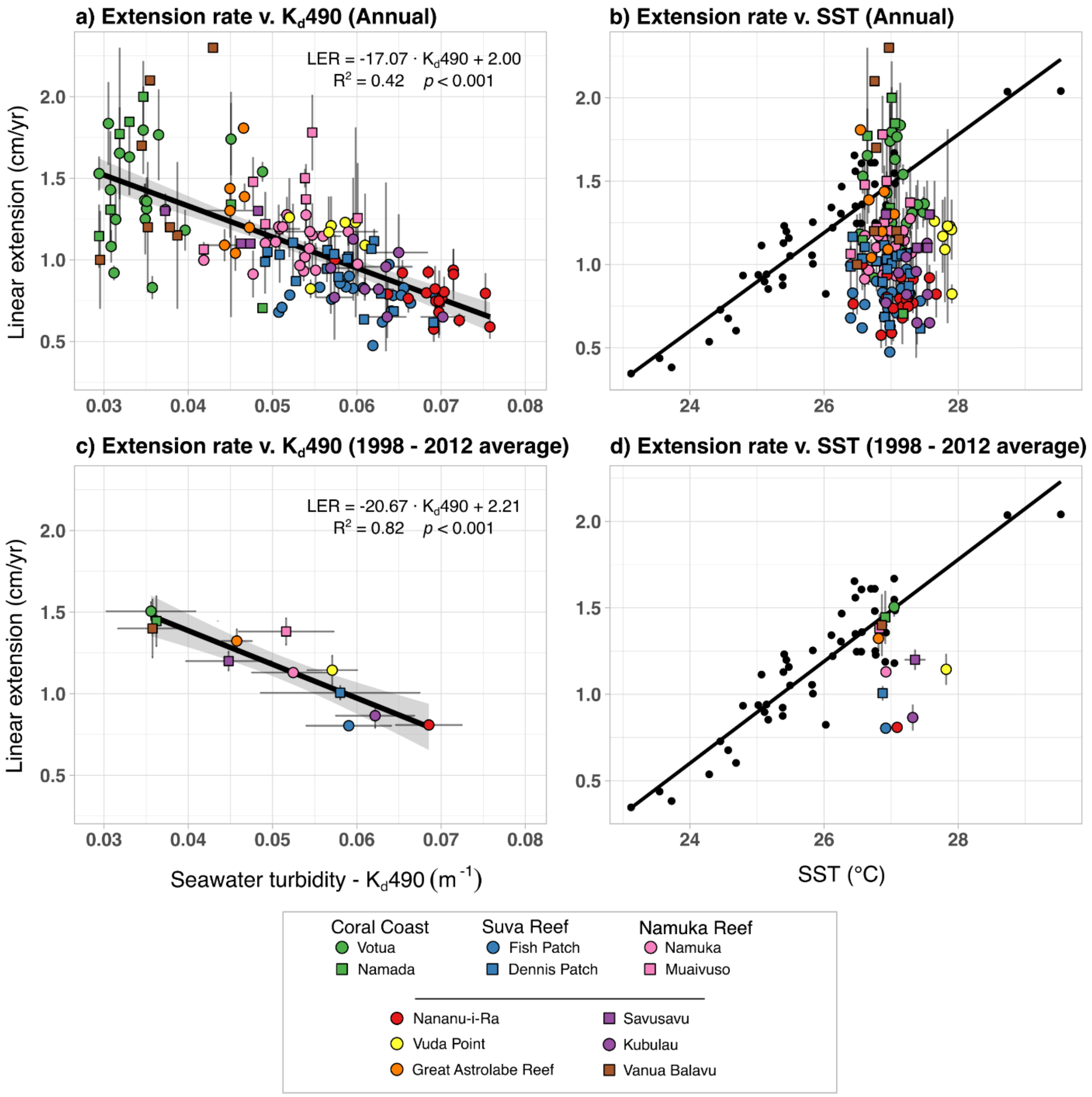

Supplementary Figure S3. Average linear extension rates at each location of this study. A) and b: annual linear extension v. seawater turbidity (measured as K_d490) and SST, respectively. C) and d): location mean linear extension for the 1998 – 2012 period (no thermal stress). V. seawater turbidity and SST. Averages are obtained from the coral cores collected at each location. Error bars reflect SE. A significant relationship is only observed between linear extension and seawater turbidity. Black solid lines in a) and c) represent the linear relationship with 95% confidence interval (grey area). Black datapoints and black line in b) and d) are extracted from Lough et al. 2014 and represent core average data from Porites spp. in the Great Barrier Reef and their relationship with SST.

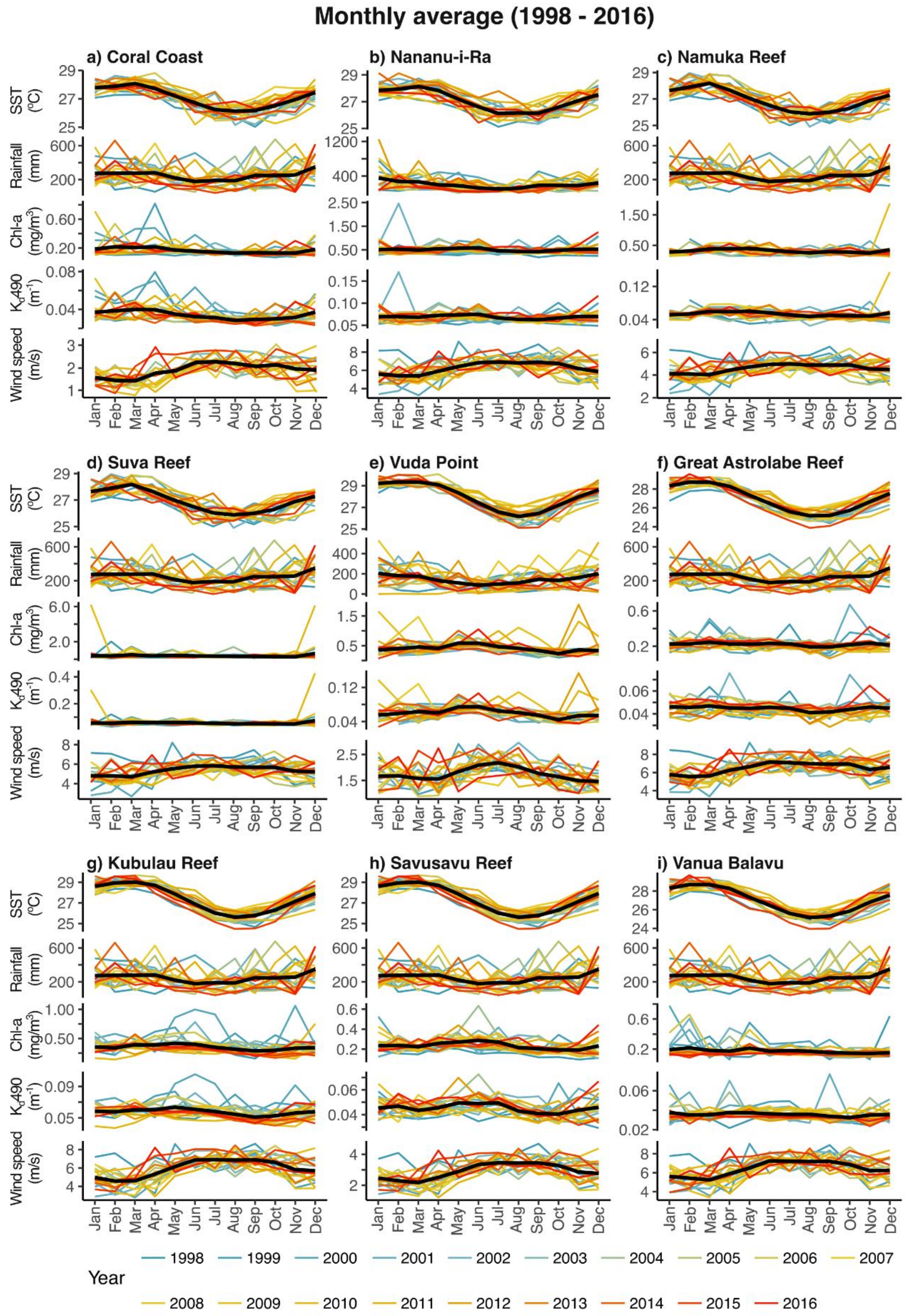

Supplementary Figure S4. 1998 – 2016 monthly mean data for all environmental variables at every location were samples included here were collected. Black line represents the climatological mean for each month.

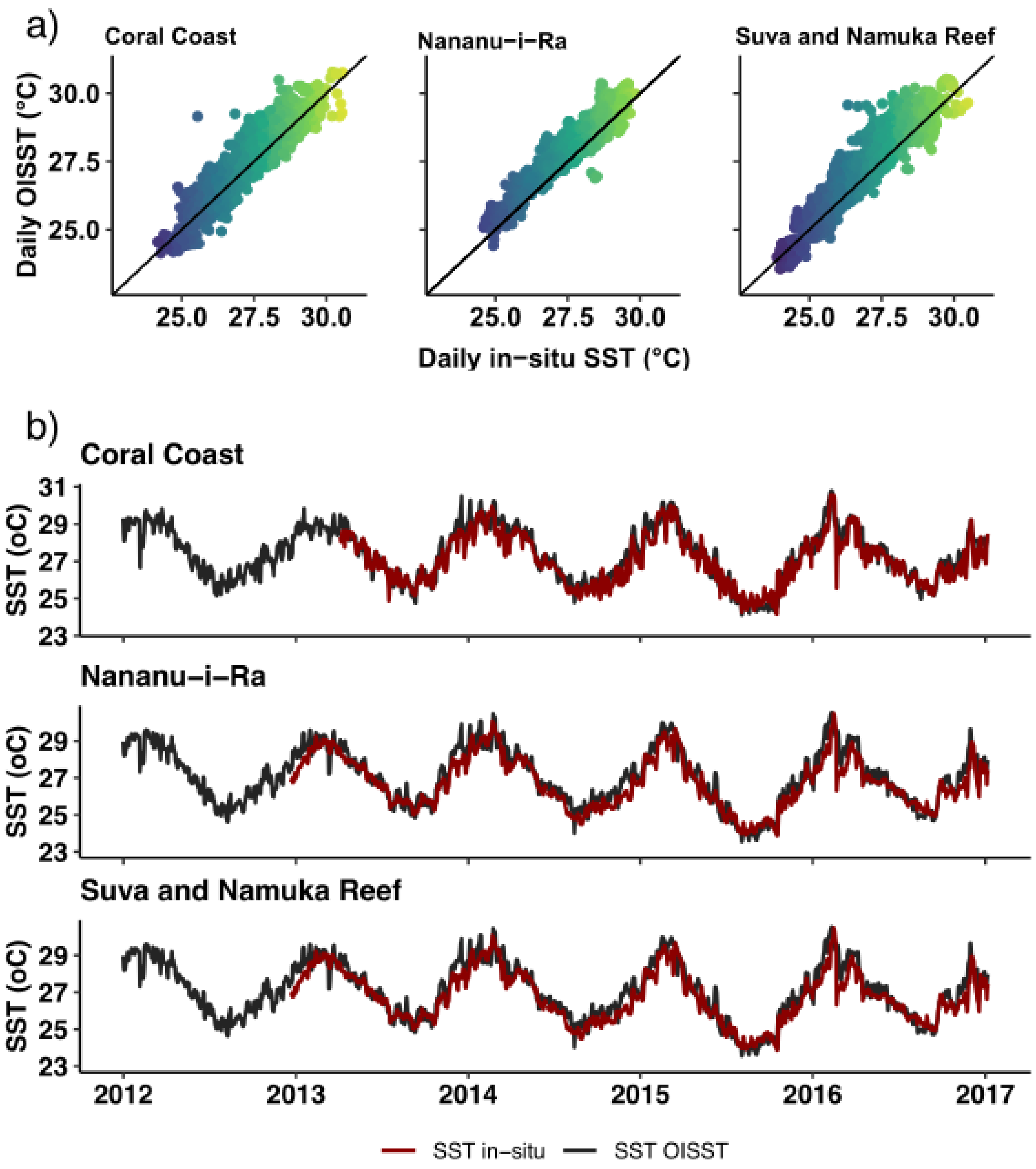

Supplementary Figure S5. OISST data validation with *in situ* seawater temperature loggers. a) Scatterplot of daily *in situ* seawater temperature data and remotely sensed SST from OISST. Black line represents the 1:1 correlation line. b) Daily SST data at each location from *in situ* loggers (red line) and OISST product (grey line). Only three locations were included in the SST validation (Coral Coast, Nananu-i-Ra and Suva and Namuka Reefs) because these were the only locations of this study with nearby *in situ* loggers available before 2017.

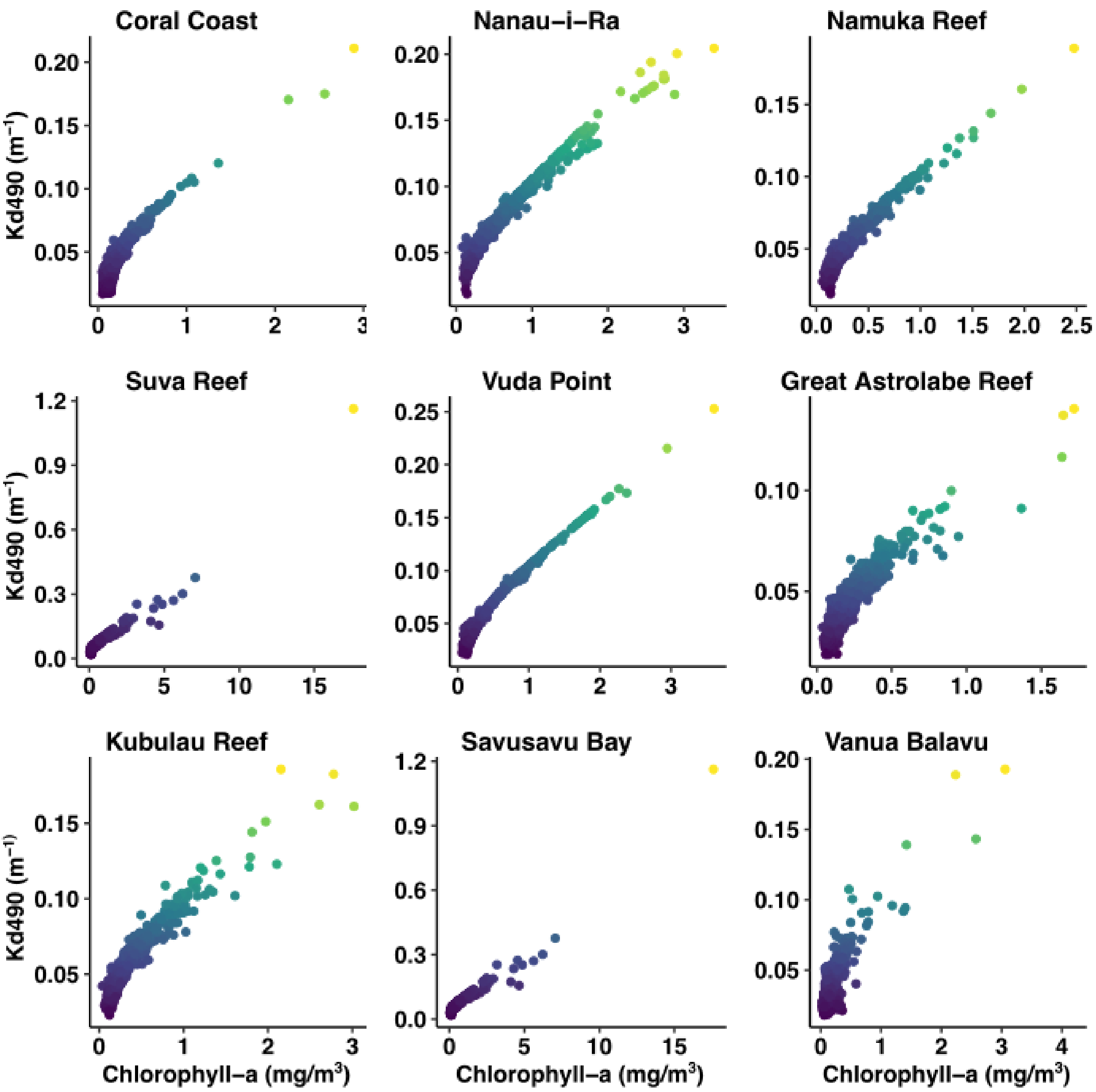

Supplementary Figure S6. Linear correlation between daily Chlorophyll-a and K_d490 (Ocean Color Data product) at each location of this study.

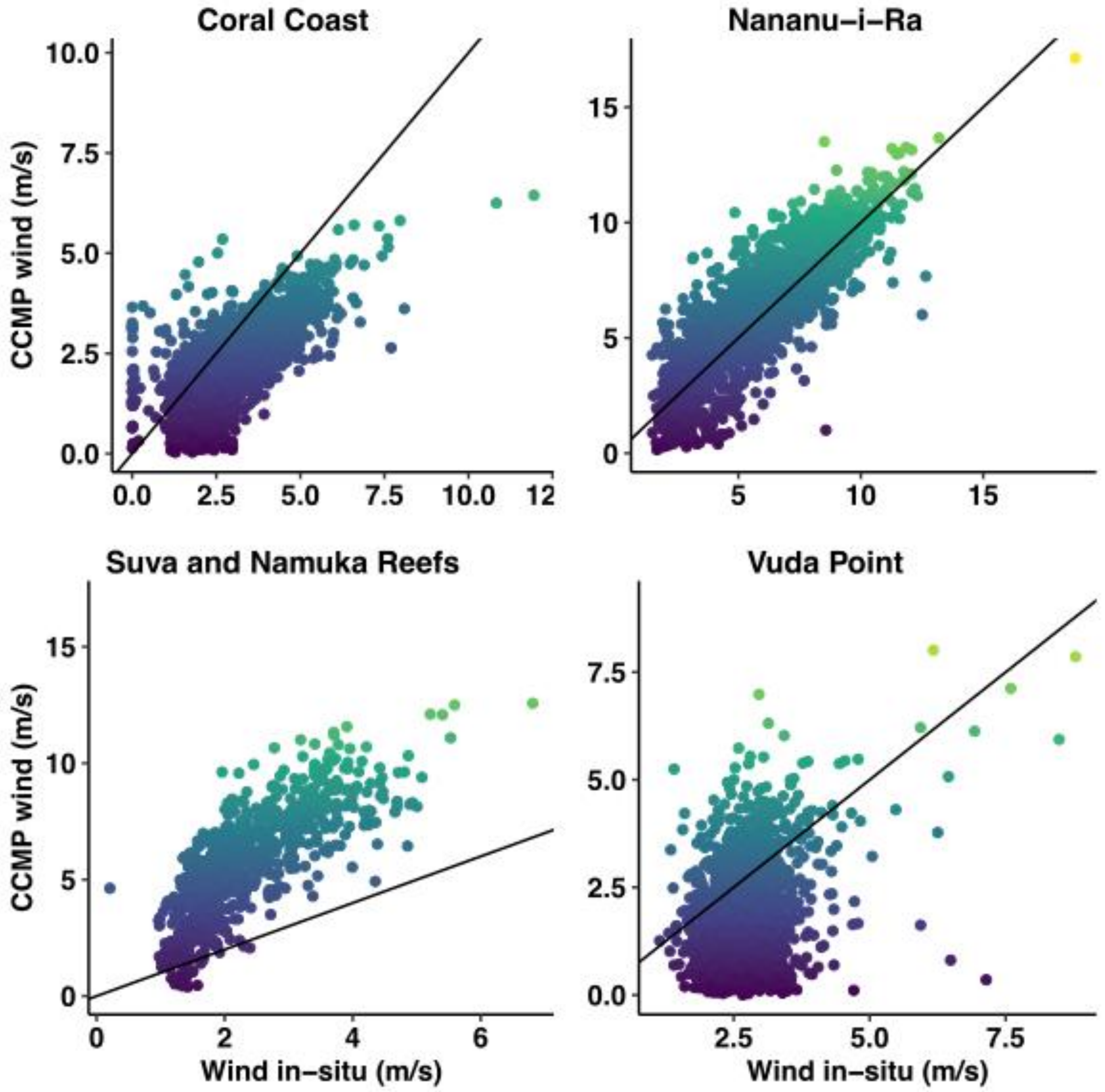

Supplementary Figure S7. Scatterplot of wind speed data from available local weather stations (*in situ*) and remotely sensed data from CCMP product. Black line represents the 1:1 correlation line.

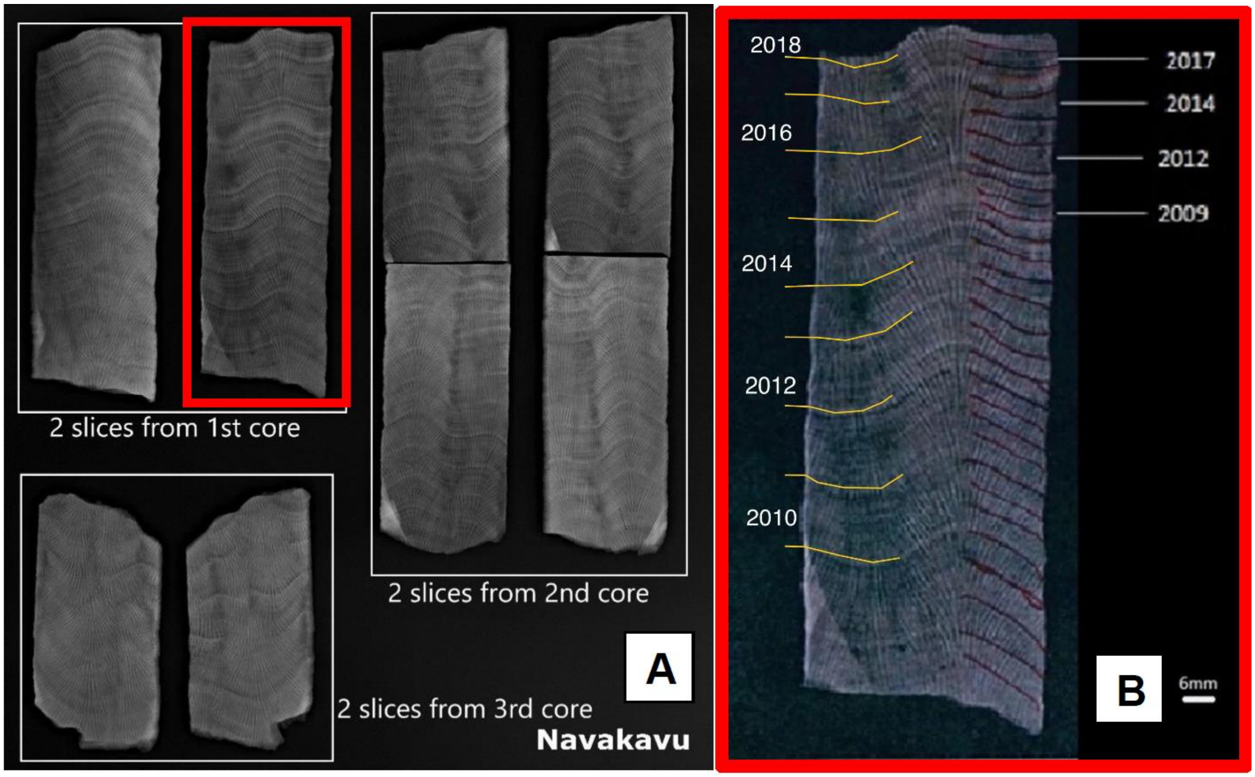

Supplementary Figure S8. Age model correction from samples in Goberdhan and Kinninmonth (2021). X-ray slabs from a *Porites* spp. collected in Navakavu (Namuka Reef) and published in Goberdhan and Kinninmonth (2021). A) Shows all the slabs and sections from the collected core. B) is close-up from slab within red rectangle. Core chronology on the right (white and black lines) is the age model built by Goberdhan and Kinninmonth (2021). Core chronology on the left (yellow lines) is the reinterpreted age model and used in this study. The same protocol was followed for all the cores from Goberdhan and Kinninmonth (2021) used in this study after obtaining X-rays and age model from the authors. Reinterpreted age models of other samples are available upon request, due to these not being publicly available.

Supplementary Text S1: Environmental data

Below are detailed the source and methods for obtaining environmental data in this study. Supplementary Figure S4 presents monthly values for each variable and location. Supplementary Table S1 details the coral coring coordinates and locations.

1. Seawater surface temperature (SST) and thermal stress

Gridded satellite-derived seawater surface temperature (SST) was obtained for each location to obtain a complete dataset covering at least the same period as coral data studied here (1998 – 2016) from Daily Optimum Interpolation SST (OISST)[5,6]. SST data was extracted from the single pixel containing the core location, or the closest available. OISST dataset is available daily (nighttime) with a resolution of ~25 km. This dataset combines satellite products derived from AVHRR sensors and *in situ* observations (from buoys and ships). Different SST gridded products have been used in coral research. The different algorithms applied to obtain SST from satellite data, and the different spatial resolution and coverage can result in potential regional biases in the different products, making some datasets more accurate in certain locations and timeframes. To validate this SST satellite product, we validated the data with *in situ* available seawater temperature data.

In situ local seawater temperature was obtained from the ReefTEMPS network dataset (Varillon et al. 2018). ReefTEMPS encompasses a network of seawater sensors in the South and West Pacific Ocean. Of these networks, a total of 11 coastal sensors are anchored in Fijian marine territory, of which four are located near locations in this study (i.e. Coral Coast, Namuka Reef, Suva Reefs, and Nananu-i-Ra in Viti Levu). Temperature data from the closest available logger to each location of this study is used. Temporal resolution of raw data is 10 minutes, covering from ~2013 to May 2017 (date of core collection). Data reduction was carried out to obtain a daily resolved dataset by calculating a mean average.

Mean remotely sensed SST showed no clear differences between satellite products or with *in situ* data (Supplementary Table S4). In addition, paired correlations of remotely sensed and *in situ* data show high degrees of correlations at all locations ($R^2 > 0.93$) (Supplementary Figure S5). This indicates that remotely sensed SST can represent *in situ* seawater temperature well in all inshore reefs of this study.

Thermal stress was evaluated as Degree Heating Weeks (DHW). DHW were obtained from the CRW *CoralTemp*'s daily global 5 km satellite coral bleaching DHW[7]. This product measures the accumulation of heat stress that coral reefs experienced over the prior 12 weeks. It is a cumulative measurement of both intensity and duration of heat stress, and it is expressed in the unit °C-weeks. DHWs over 4 °C-weeks have been shown to cause significant coral bleaching; values over 8 °C-weeks have caused severe bleaching and significant mortality.

2. Seawater turbidity and chlorophyll-a concentration

To characterise water quality at each location, the diffuse attenuation coefficient at a wavelength of 490 nm ($K_d490[m^{-1}]$) and the chlorophyll-a concentration (mg/m^3) were derived from satellite ocean colour (OC) data[8]. Level-3 data (gridded) were obtained from both SeaWiFS[9] and MODIS-Aqua[10] products. SeaWiFS product is available with a resolution of 9 km, and daily data was obtained between September 1997 and July 2002. The MODIS-Aqua product has a resolution of 4 km, and daily data were obtained between July 2002 until May 2017. Similar to SST, an area of 1x1 pixel enclosing each core location was selected.

Across all four locations of this study K_d490 and chlorophyll-a values show a high degree of correlation ($R^2 = 0.93$, $p < 0.0001$) (Supplementary Figure S6). This observation is not surprising, as turbid water can be associated with biological processes such as phytoplankton blooms (e.g. Platt et al.[11]) and/or river runoff and associated sediment plumes[12], and sediment resuspension and transportation [13,14]. Supporting this observation, Shi and Wang[8] found that moderate increases in seawater turbidity ($K_d490 < 0.3$ m^{-1}), like those observed across all locations here, tend to be associated with an elevation of chlorophyll-a concentration. Considering this, and the close correlation between the two variables, in this study we use K_d490 in the figures and representations as a more comprehensive variable, although both variables were statistically tested at all times.

3. Rainfall

Rainfall data for this study was provided by the Fiji Meteorological Service. Only rainfall data from two stations (Laucala Bay [Southern Viti Levu] and Penang Mill [Northern Viti Levu]) was available covering at least the 1998 – 2017 period. Laucala Bay was paired with growth data from locations in Southern Viti Levu (i.e. Coral Coast, Namuka and Suva Reefs), and Penang Mill was paired with coral data from Nananu-i-Ra Reef, in Northern Viti Levu. Data was available as daily accumulated rainfall (mm). For this study, we used both monthly accumulated rainfall and monthly mean, as well as annual mean.

4. Wind speed

Daily *in situ* wind direction and speed was also provided by the Fiji Meteorological Service from two locations, Laucala Bay (Southern Viti Levu) and Rakiraki (Northern Viti Levu). Length of datasets was 2015 – 2017 and 2013 – 2017 respectively. As with rainfall data, Laucala Bay data was coupled with Southern Viti Levu locations (i.e. Coral Coast, Namuka and Suva Reefs), and Rakiraki wind data was coupled to Nananu-i-Ra Reef (Northern Viti Levu) variables.

Satellite wind data was obtained from the Cross-Calibrated Multi-Platform (CCMP), a gap-free gridded product produced using satellite and buoy wind measurements. The available resolution of data is 6-hourly at ~25 km. CCMP v 2.0 data was retrieved from 1x1 pixel encompassing each coral location from January 2002 until December 2016 (end of dataset).

To validate satellite observations, a validation with the available *in situ* data was carried out. Mean wind data is similar between remotely sensed data and *in situ* data in both the Coral Coast and Nananu-i-Ra (Supplementary Table S4). However, notable differences are seen for the Namuka reef data ($\Delta = 1.49$ m/s) and Suva Reef ($\Delta = 3.01$ m/s). Paired correlations show high correlation of data (Supplementary Figure S7); however, data shows large scatter around the 1:1 line across the entire range of wind speeds, as well as a positive bias for remote sensed data in both Namuka and Suva Reef, where satellite product show higher wind speeds than *in situ* data from Laucala Bay. Wind speed conditions tend to be highly localised and can vary greatly across short distances, especially due to orographic effects on flow dynamics. *In situ* values are measured in-land, while satellite values are taken from pixel data above the coast/inshore reefs, and therefore a difference in winds is to be expected. These results indicate that remotely sensed wind data can be representative of general conditions in the study locations, but absolute values should be interpreted with caution.

Supplementary references

1. Jupiter, S. et al. Integrating EBM Science to Assess Marine Protected Area Effectiveness: Clues from Coral Proxies of Land Disturbance, Ecological Assessments and Socioeconomic Surveys. (2010).
2. Goberdhan, L. & Kininmonth, S. Insights into coral growth rate trends in Fiji. *Coral Reefs* **40**, 251–266 (2021).
3. Linsley, B. K. et al. Tracking the extent of the South Pacific Convergence Zone since the early 1600s. *Geochem. Geophys. Geosyst.* **7**, Q05003 (2006).
4. Dassié, E. P. et al. A Fiji multi-coral δ18O composite approach to obtaining a more accurate reconstruction of the last two-centuries of the ocean-climate variability in the South Pacific Convergence Zone region. *Paleoceanography* **29**, 1196–1213 (2014).
5. Reynolds, R. W. et al. Daily High-Resolution-Blended Analyses for Sea Surface Temperature. *J. Clim.* **20**, 5473–5496 (2007).
6. Huang, B. et al. Improvements of the Daily Optimum Interpolation Sea Surface Temperature (DOISST) Version 2.1. *J. Clim.* **34**, 2923–2939 (2021).
7. NOAA Coral Reef Watch. NOAA Coral Reef Watch Version 3.1 Daily Global 5km Satellite Coral Bleaching Degree Heating Week Product. (2018).
8. Shi, W. & Wang, M. Characterization of global ocean turbidity from Moderate Resolution Imaging Spectroradiometer ocean color observations. *J. Geophys. Res. Oceans* **115**, C11022 (2010).
9. NASA Ocean Biology Processing Group. OrbView-2 SeaWiFS Global Mapped Diffuse Attenuation Coefficient for Downwelling Irradiance (KD) Data, version R2022.0. NASA Ocean Biology Distributed Active Archive Center https://doi.org/10.5067/ORBVIEW-2/SEAWIFS/L3M/KD/2022 (2022).
10. NASA Ocean Biology Processing Group. Aqua MODIS Level 3 Binned Downwelling Diffuse Attenuation Coefficient Data, Version R2022.0. NASA Ocean Biology Distributed Active Archive Center https://doi.org/10.5067/AQUA/MODIS/L3B/KD/2022 (2022).
11. Platt, T., Sathyendranath, S., Caverhill, C. M. & Lewis, M. R. Ocean primary production and available light: further algorithms for remote sensing. *Deep Sea. Res. A: Oceanogr. Res. Pap.* **35**, 855–879 (1988).
12. Nezlin, N. P. & DiGiacomo, P. M. Satellite ocean color observations of stormwater runoff plumes along the San Pedro Shelf (southern California) during 1997–2003. *Cont. Shelf Res.* **25**, 1692–1711 (2005).
13. Acker, J. G., Brown, C. W., Hine, A. C., Armstrong, E. & Kuring, N. Satellite remote sensing observations and aerial photography of storm-induced neritic carbonate transport from shallow carbonate platforms. *Int. J. Remote Sens.* **23**, 2853–2868 (2002).
14. Shi, W. & Wang, M. Three-dimensional observations from MODIS and CALIPSO for ocean responses to cyclone Nargis in the Gulf of Martaban. *Geophys. Res. Lett.* **35**, L21603 (2008).
